# The Effect of CHIR 99021, a Glycogen Synthase Kinase-3β Inhibitor, on Transforming Growth Factor β-Induced Tenon Fibrosis

**DOI:** 10.1167/iovs.62.15.25

**Published:** 2021-12-23

**Authors:** Sang Yeop Lee, Min Kyoung Chae, Jin Sook Yoon, Chan Yun Kim

**Affiliations:** 1 Department of Ophthalmology, Severance Hospital, Institute of Vision Research, Yonsei University College of Medicine, Seoul, Korea; 2 Department of Ophthalmology, Yongin Severance Hospital, Yonsei University College of Medicine, Yongin, Gyeonggi-do, Republic of Korea

**Keywords:** human tenon fibroblast, glycogen synthase kinase-3β, transforming growth factor β, CHIR 99021, bleb fibrosis

## Abstract

**Purpose:**

This study investigated the effect of glycogen synthase kinase-3β (GSK-3β) inhibition on the fibrosis of human Tenon's fibroblasts (HTFs) induced by transforming growth factor-β (TGF-β).

**Methods:**

Quantitative real-time PCR and Western blot analyses were performed to determine the expression levels of molecules associated with the fibrosis of HTFs by TGF-β (fibronectin, collagen Iα, and α-smooth muscle actin) and GSK-3β. The levels of phosphorylated Smad2 and Smad3 were also analyzed in the presence of the GSK-3β inhibitor CHIR 99021. The wound healing assay was performed to determine the effect of CHIR 99021 on the migration of HTFs. All experiments were conducted using primary cultured HTFs or human tenon tissues obtained from normal subjects and patients with glaucoma.

**Results:**

Treatment with TGF-β resulted in an increase in the levels of molecules associated with the fibrosis of HTFs. The expression levels of these molecules were higher in the tenon tissues obtained from patients with glaucoma than those from normal subjects. When the HTFs were treated with TGF-β, a significant increase in the active form of GSK-3β (Y216) was observed. A significant decrease in the active form of GSK-3β and molecules associated with fibrosis by TGF-β was noted in HTFs treated with CHIR 99021. CHIR 99021 treatment reduced the phosphorylated Smad2/Smad2 and phosphorylated Smad3/Smad3 ratios in HTFs and attenuated HTF migration.

**Conclusions:**

Our results demonstrated the effect of GSK-3β inhibition on the regulation of TGF-β–mediated fibrosis of HTFs, suggesting GSK-3β to be a potential target for maintaining bleb function after glaucoma filtration surgery.

Glaucoma is a progressive optic neuropathy characterized by the loss of retinal ganglion cells, resulting in morphological changes in the optic nerve head and associated visual field defects.^1^ Because IOP is the only known modifiable risk factor in glaucoma, its treatment focuses on the decrease of IOP.^2^ Glaucoma filtration surgery, which provides an alternative route for the outflow of aqueous humor from the anterior chamber, is a widely used surgical method for the effective decrease of the IOP. The success of a glaucoma filtration surgery depends on the maintenance of a functioning bleb, which is the area underneath the conjunctiva and Tenon's capsule, because it plays a role in controlling the efflux of aqueous humor from the anterior chamber.^3^ However, there is always a risk of excessive bleb fibrosis, which interrupts the flow of aqueous humor during postoperative healing.

Myofibroblast transdifferentiation of human Tenon's fibroblasts (HTFs) induces subconjunctival fibrosis by triggering the production of extracellular matrix proteins and enhancing contractile activity, with subsequent hypertrophic scarring.^3^^,^^4^ Hence, appropriate control of this process would increase the success rate of glaucoma filtration surgery by preventing excessive bleb fibrosis. Transforming growth factor-β (TGF-β) plays an important role in myofibroblast transdifferentiation.^5^^,^^6^ Therefore, inhibition of the TGF-β signaling pathway could help prevent HTF activation. Clinical studies have investigated the antibleb fibrosis effect of CAT-152 (lerdelimumab), a monoclonal antibody of TGF-β2, or ISTH0036, an antisense oligodeoxynucleotide targeting TGF-β2.^7^^–^^9^ In the case of CAT-152, a phase III study was conducted, but compared with the placebo, there was no significant difference in the prevention of trabeculectomy failure.^8^ The following factors are thought to be related to the failure of phase III clinical trials. First, CAT-152 was not administered at a sufficient dose or for a sufficient period of time owing to differences in TGF-β levels among individuals. Next, nonstandardization of postsurgical intervention in the participating institutions possibly led to the high success rate of the placebo group. In addition, the effect of other TGF-β isoforms on scar formation was not considered in that study. A clinical study using ISTH0036 obtained positive results,^9^ but additional clinical studies are needed. Given that activation of the TGF-β signaling pathway is necessary for the maintenance of a nonpathologic status,^10^ the corresponding risk of blockade should be considered while regulating this pathway. Hence, it would be more advantageous to control the downstream signaling pathway of TGF-β, while maintaining the role of TGF-β in normal healing.

Glycogen synthase kinase-3 (GSK-3) is a protein–serine kinase that lies downstream of multiple signaling pathways.^11^ Previous studies have demonstrated the crosstalk between GSK-3 and TGF-β during multiple organ fibrosis, with controversial results. The inhibition of GSK-3β attenuates the process of pulmonary fibrosis^12^^,^^13^ or renal fibrosis,^14^^,^^15^ while increasing myocardial fibrosis.^16^^,^^17^ These results indicate that GSK-3β likely plays an important role in the regulation of fibrosis. However, the role of GSK-3 in the process of bleb fibrosis in glaucoma filtration has not been explored. Hence, in the present study, we investigated the role of GSK-3β in TGF-β–related fibrosis of HTFs using the GSK-3β inhibitor CHIR 99021.

## Methods

### Reagents

CHIR 99021 was obtained from Tocris Bioscience (Minneapolis, MN). The 3-(4,5-dimethylthiazol-2-yl)-5-(3-carboxymethoxyphenyl)-2-(4-sulfophenyl)-2H-tetrazo-lium (MTT) assay kit was purchased from Sigma-Aldrich (St. Louis, MO). Fetal bovine serum, Dulbecco's modified Eagle's medium (DMEM), penicillin, and streptomycin were purchased from Hyclone Laboratories, Inc. (Logan, UT). The antibodies for phosphorylated GK-3β (S9), GSK-3β, β-catenin, nonphosphorylated β-catenin, Smad2, phosphorylated Smad2 (Ser465/467), Smad3 (Ser423/425), and phosphorylated Smad3 were purchased from Cell Signaling Technology (Beverly, MA). The antibodies for phosphorylated GSK-3β (Y216) and fibronectin were purchased from BD Bioscience (San Jose, CA). Collagen Iα and β-actin antibodies were purchased from Santa Cruz Biotechnology (Santa Cruz, CA). The antibody for α-smooth muscle actin (α-SMA) was purchased from Sigma-Aldrich.

### Subjects and Preparation of Tissues and Cells

The human subconjunctival Tenon's capsules were obtained with written informed consent from subjects undergoing cataract or glaucoma filtration surgery during the process of sub-Tenon anesthesia or detachment of the subconjunctival space, at the glaucoma clinic of Yonsei University Severance Hospital, Seoul, Korea. The subjects were five patients without glaucoma undergoing cataract surgery (one man and four women; mean age, 62.4 ± 5 years) and five patients with glaucoma undergoing cataract surgery or cataract and Ahmed valve insertion combined surgery (one man and four women; mean age, 70.2 ± 9.6 years). The study protocol was reviewed and approved by the Institutional Review Board of Severance Hospital (4-2020-0637). The study adhered to the tenets of the Declaration of Helsinki.

HTFs were isolated as previously described.^18^ The characteristics of the patients included in the study are detailed in Supplementary Table S1. The tissues were cut into 1- to 2-mm pieces under sterile conditions using a stereo microscope. The sample was minced and placed in DMEM/F12 (1:1 ratio) medium supplemented with 10% fetal bovine serum, 100 U/mL penicillin, and 100 µg/mL streptomycin at 37 °C and 5% CO_2_. When the growth of the fibroblasts was well-established, the cells were treated with trypsin/EDTA and passaged in a monolayer. We used only those cells that were between the third and fifth passages for experiments. Except for the comparison of mRNA levels for molecules associated with TGF-β–induced fibrosis and GSK-3β expression levels between subjects with and without glaucoma, all experiments were conducted using fibroblasts obtained from subjects without glaucoma.

### Cell Viability Assay

The MTT assay was performed to assess the effect of CHIR 99021 on cell viability. As per the manufacturer's protocol (Sigma-Aldrich), HTFs were seeded into 24-well culture plates (1 × 10^5^ cells/well) and treated with different concentrations of CHIR 99021 (1, 2.5, 5, 10, and 20 µM) and dimethylsulfoxide (DMSO) for 24, 48, and 72 hours. Then, cells were washed and incubated in 5 mg/mL MTT solution for 3 hours at 37 °C. After solubilization following the addition of ice-cold isopropanol, the absorbance of the converted dye was measured using a microplate reader (EL 340 Microplate Bio Kinetics Reader; Bio-Tek Instrument, Winooski, VT) at 560 nm, with background subtraction at 630 nm.

### Quantitative Real-time PCR

For a quantitative evaluation of the level of gene transcription, quantitative real-time PCR was performed following the manufacturer's protocol. The RNA was extracted from cells using TRIzol (Thermo Fisher Scientific, Waltham, MA). The extract was reverse transcribed into cDNA (Qiagen, Hilden, Germany). Thereafter, cDNA was amplified with SYBR Green Real-Time PCR Master Mix in a StepOne Plus real-time PCR thermocycler (Applied Biosystems, Carlsbad, CA). The sequences of PCR primers used in the experiment are presented in Supplementary Table S2. The amount for each type of mRNA normalized to the glyceraldehyde-3-phosphate dehydrogenase was calculated and expressed as fold change in the threshold cycle (Ct) value relative to the control group using the 2^−△△Ct^ method.^19^

### Western Blotting

After washing with PBS, HTFs were lysed with cell lysis buffer on ice. The lysates were centrifuged at 15,000 *× g* for 10 minutes at 4 °C. The supernatants were resolved via 10% sodium dodecyl sulphate-polyacrylamide gel electrophoresis and the separated proteins were transferred onto polyvinylidene fluoride membranes (Immobilon; Millipore Corp., Billerica, MA). The membranes were probed with primary antibodies in Tris-buffered saline containing Tween 20, 0.001% sodium azide, and 1% BSA. Subsequently, horseradish peroxidase-conjugated secondary antibody with chemiluminescence (Cell Signaling Technology) was used for probing these membranes. The bands were detected on x-ray films (Agfa-Gevaert, Mortsel, Belgium). β-Actin was used as the loading control and to normalize the intensity of the band detected for each sample.

### Wound Healing Assay

HTFs were seeded in 6-well plates and grown to 100% confluence. The surface of the single layer of cells was scratched using a 200-µL pipette tip to create a vertical wound. The cells were then washed with PBS, after which fresh DMEM/F12 containing DMSO or different concentrations of CHIR 99021 (1 µM, 5 µM, and 10 µM) was added. The plates were incubated for 24 hours and 48 hours, and the distance of cell migration was measured using a phase-contrast microscope (×1.5 magnification).

### Statistical Analyses

All the experiments were conducted in triplicate, and data were expressed as mean ± standard deviation. Independent *t*-test and ANOV were used for comparisons between groups. Post hoc tests were conducted using Bonferroni analysis. SPSS for Windows, version 25 (SPSS, Inc., Chicago, IL) was used. A *P* value of less than 0.05 was considered to be statistically significant.

## Results

### Viability of HTFs

**Figure 1.**
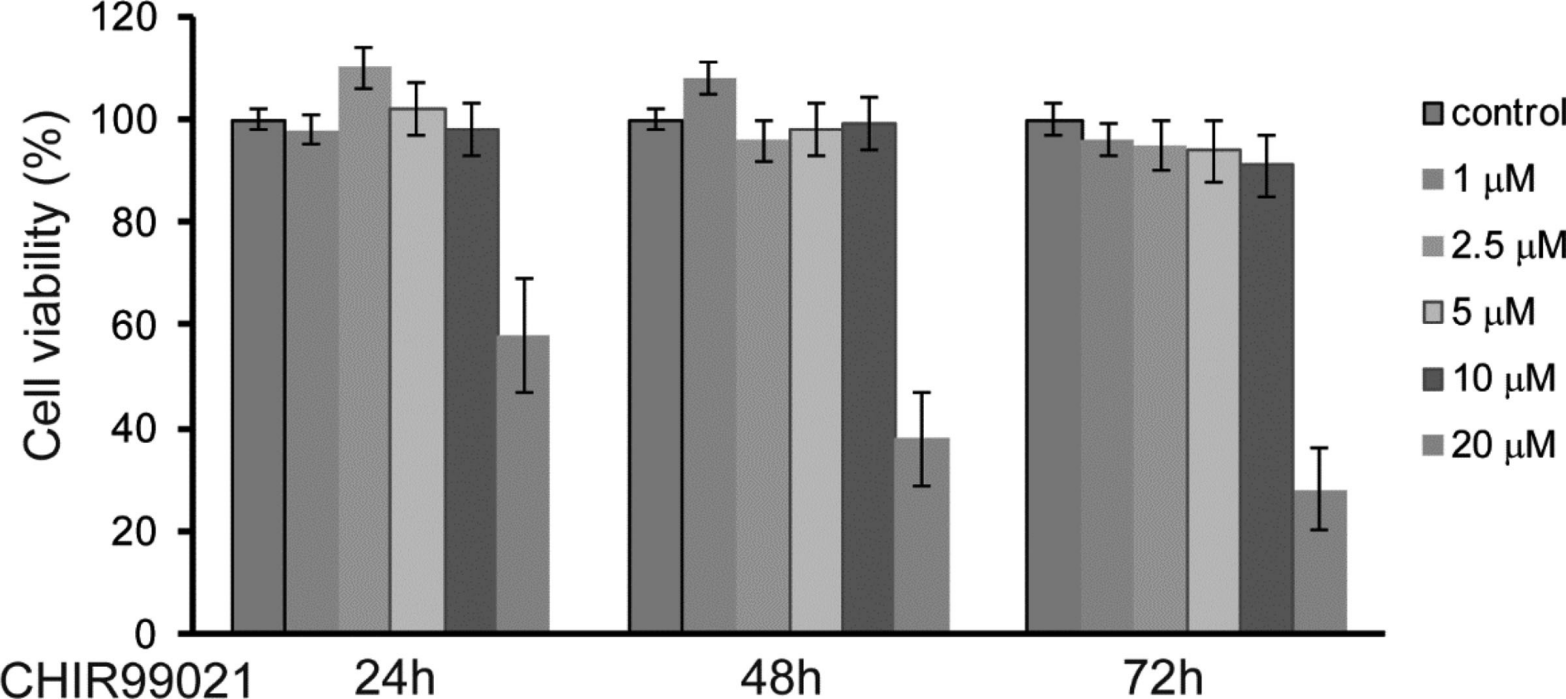
Effect of CHIR 99021 on the viability of HTFs. HTFs were seeded in 24-well culture plates (1 × 10^5^ cells/well). Different concentrations of CHIR 99021 (ranging from 1 µM to 20 µM) were applied to the wells for 24, 48, and 72 hours. An MTT assay was used for determining cell viability.

HTFs were treated with various doses of CHIR 99021 for 24, 48, and 72 hours. The number of live cells were determined using the MTT assay. The cell viability was close to 100% at all exposure times, except at a concentration of 20 µM CHIR 99021 (Fig. 1). Based on this experiment, we decided on using of CHIR 99021 at a concentration of 5 or 10 µM in subsequent experiments.

### Increased Expression of GSK-3β in HTFs Activated by TGF-β

**Figure 2.**
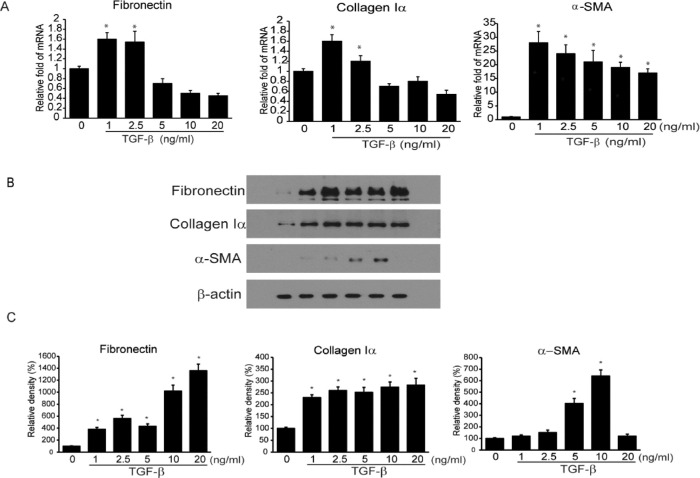
HTFs were treated with various concentrations of TGF-β (1, 2.5, 5, 10, and 20 ng/mL) for 24 hours. The mRNA (A) and protein (B and C) levels of fibronectin, collagen Iα, and α-SMA were analyzed via quantitative reverse transcription PCR and Western blotting, respectively. The results are presented as the mean ± standard deviation of three separate experiments (*P < 0.05 compared with control).

HTFs were incubated with various concentrations (0, 1, 2.5, 5, 10, and 20 ng/mL) of TGF-β for 24 hours. Because fibrosis is characterized by the upregulation of fibronectin, collagen Iα, and α-SMA expression, the changes in the mRNA and protein expression levels for these molecules were evaluated using real-time PCR and Western blotting (Fig. 2). In real-time PCR, the transcription of α-SMA showed a marked increase at all concentrations of TGF-β, compared with the control, whereas that of fibronectin and collagen Iα showed a significant increase at TGF-β concentrations of 1.0 and 2.5 ng/mL (Fig. 2A). As per Western blotting results (Fig. 2B and 2C), with an increase in the concentration of TGF-β, there was a significant increase in the expression levels of fibronectin and collagen Iα proteins. The expression of α-SMA protein also increased significantly at 5 and 10 mg/mL of TGF-β.

We compared the levels of fibronectin, collagen Iα, α-SMA, and GSK-3β between tenon tissues in normal subjects and patients with glaucoma (Supplementary Fig. S1). All molecules capable of activating HTFs (fibronectin, collagen Iα, and α-SMA) showed significantly higher levels of mRNA expression in glaucoma patients compared with normal subjects. Although not statistically significant, the mRNA expression of GSK-3β was also elevated in patients with glaucoma.

**Figure 3.**
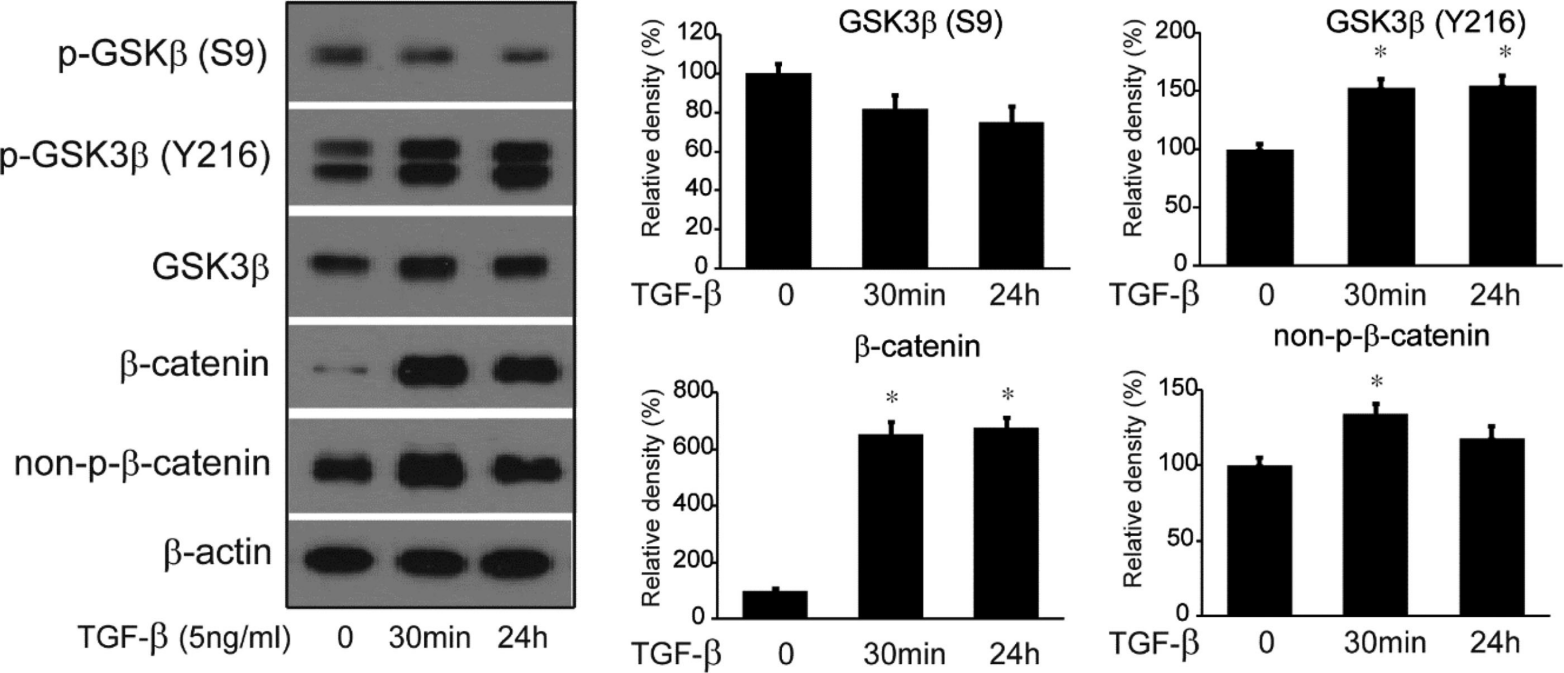
TGF-β increases levels of active form of GSK-3β (phosphorylation of Y216 residue) and activates β-catenin signaling pathway in HTFs. When TGF-β was applied to HTFs at different time points (0 minutes, 30 minutes, and 24 hours), the level of the active form of phosphorylated GSK-3β (Y216) increased, significantly. However, the level of inactive form of GSK-3β (S9) decreased. Increasing durations of TGF-β treatment resulted in increased level of total β-catenin and nonphosphorylated β-catenin (active form of β-catenin). Data in the columns indicate the mean relative density ratio ± standard deviation of three separate experiments, normalized to the level of β-actin in the same sample. Differences between untreated and treated cells are indicated (*P < 0.05).

The treatment of HTFs with TGF-β (5 ng/mL) led to a significant increase in the production of the active form of GSK-3β (phosphorylation of Y216 residue) and a decrease in the production of the inactive form of GSK-3β (phosphorylation of S9 residue) for increasing lengths of time (0 to 24 hours), as identified by Western blotting (Fig. 3). The levels of β-catenin and nonphosphorylated β-catenin were also found to increase upon treatment with TGF-β.

### CHIR 99021 Inhibits GSK-3β Production in HTFs Activated by TGF-β

**Figure 4.**
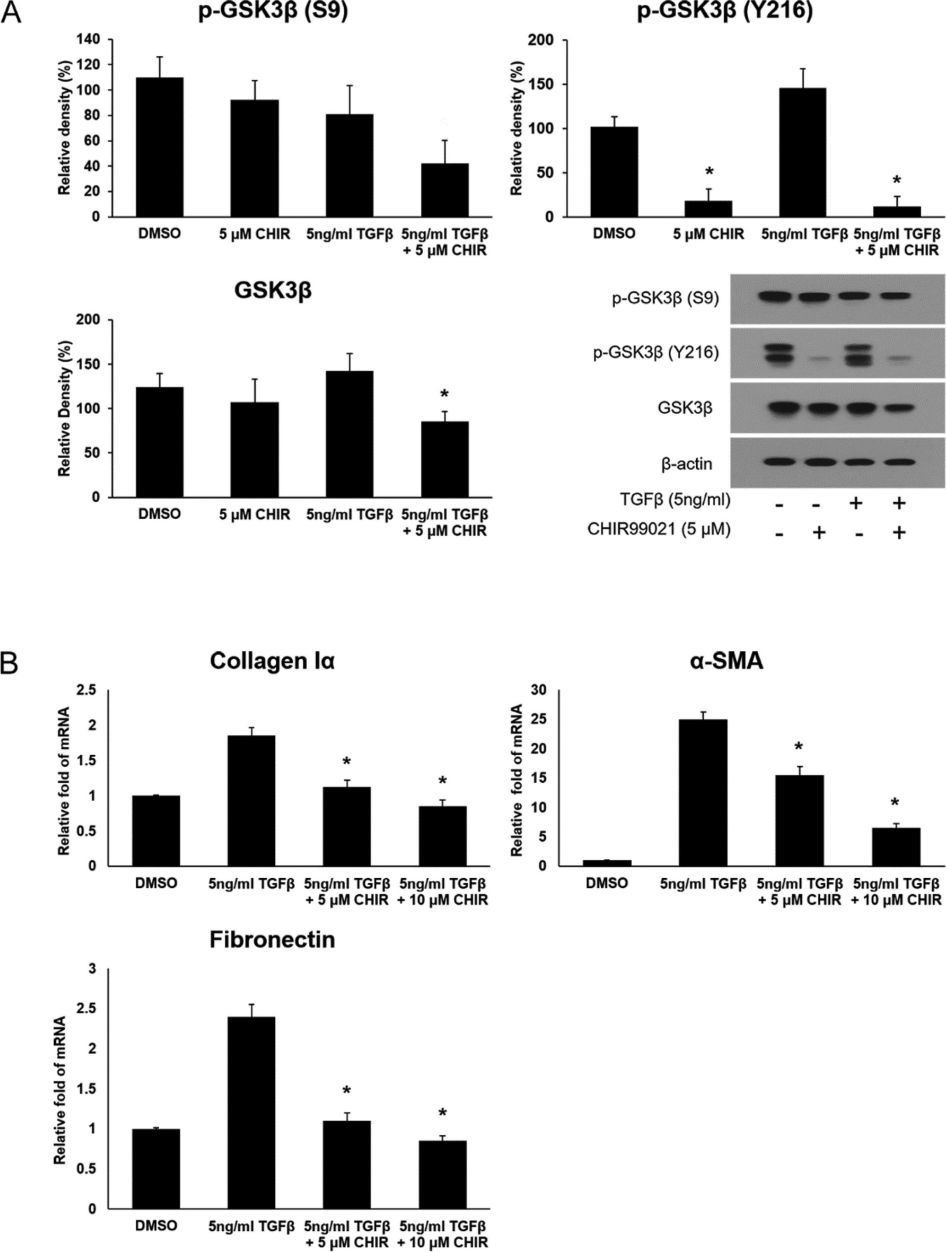
CHIR 99021 suppresses GSK-3β activation and mRNA expression of molecules associated with TGF-β–induced fibrosis in HTFs. (**A**) HTFs were treated with DMSO, 5 µM of CHIR 99021, 5 ng/mL of TGF-β, and 5 µM of CHIR 99021; then, after pretreatment with 5 ng/mL of CHIR 99021 for 48 hours, HTFs were incubated with 5 ng/mL of TGF-β for 24 hours. To evaluate the levels of the inactive form of GSK-3β [p-GSK-3β (S9)], the active form of GSK-3β [p-GSK-3β (Y216)], and total GSK-3β, Western blot analyses were performed. Treatment with CHIR 99021 resulted in a decrease in p-GSK-3β (Y216) levels, which showed an increase after treatment of TGF-β. (**B**) To investigate the effect of CHIR 99021 on the proteins associated with TGF-β-induced fibrosis, HTFs were pretreated with 5 µM and 10 µM of CHIR 99021 for 48 hours, followed by treatment with 5 ng/mL of TGF-β for 30 minutes. The increased mRNA levels of collagen Iα, α-SMA, and fibronectin, which were induced by pretreatment with TGF-β, were significantly reduced upon treatment with CHIR 99021. The extracted RNA sample was reverse transcribed and then amplified using real-time PCR, after which the mRNA level was quantified. Data in the columns indicate the mean relative density ratio or mean relative RNA fold ± standard deviation of three experiments. Differences between cells treated with TGF-β alone and both TGF-β and CHIR 99021 are indicated (*P < 0.05).

**Figure 5.**
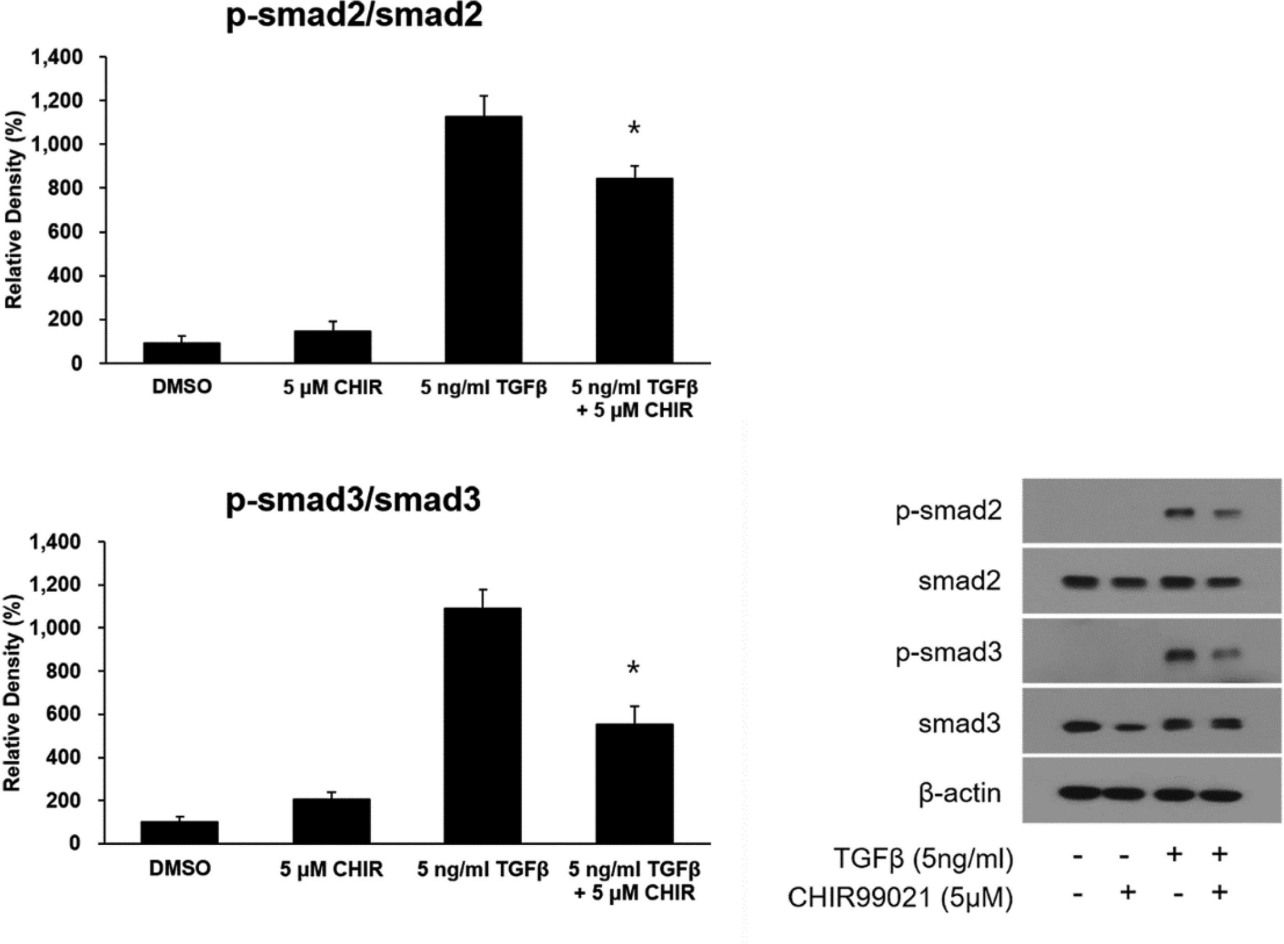
The effect of GSK-3β inhibitor on phosphorylation of Smad2 (Ser465/467) and Smad3 (Ser423/425). TGF-β caused elevated relative band densities for phosphorylated Smad2/Smad2 and phosphorylated Smad3/Smad3 ratios, which were significantly decreased on treatment with CHIR 99021. HTFs were incubated with 5 ng/mL of TGF-β for 30 minutes after pretreatment with 5 µM of CHIR 99021 for 48 hours. The data in the columns are the mean relative density ratio ± standard deviation of three experiments. Differences between cells treated with TGF-β alone and both TGF-β and CHIR 99021 are indicated (*P < 0.05).

When HTFs were treated with 5 µM of CHIR 99021, with or without the addition of 5 ng/mL of TGF-β, there was a significant decrease in the production of the active form of GSK-3β (Fig. 4A). Although the production of the inactive form of GSK-3β also decreased upon treatment with both 5 µM of CHIR 99021 and 5 ng/mL of TGF-β, it was not significant (Fig. 4A). To investigate the effect of CHIR 99021 on the molecules related to TGF-β–induced fibrosis, HTFs were pretreated with 5 µM and 10 µM of CHIR 99021 for 48 hours, followed by treatment with 5 ng/mL of TGF-β for 30 minutes. Real-time PCR elucidated a significant decrease in the mRNA expression of fibronectin, collagen Iα, and α-SMA (Fig. 4B). To further examine the effect of the GSK-3β inhibitor on the TGF-β pathway, we evaluated the activation of Smad2 and Smad3 upon treatment with TGF-β and CHIR 99021 (Fig. 5). The phosphorylated Smad2/Smad2 and phosphorylated Smad3/Smad3 ratios were elevated after treatment with 5 ng/mL of TGF-β. However, CHIR 99021 treatment attenuated the effects of TGF-β treatment, which had led to a significant increase in the phosphorylated Smad2/Smad2 and phosphorylated Smad3/Smad3 ratios.

### CHIR 99021 Suppressed Migration of HTFs

**Figure 6.**
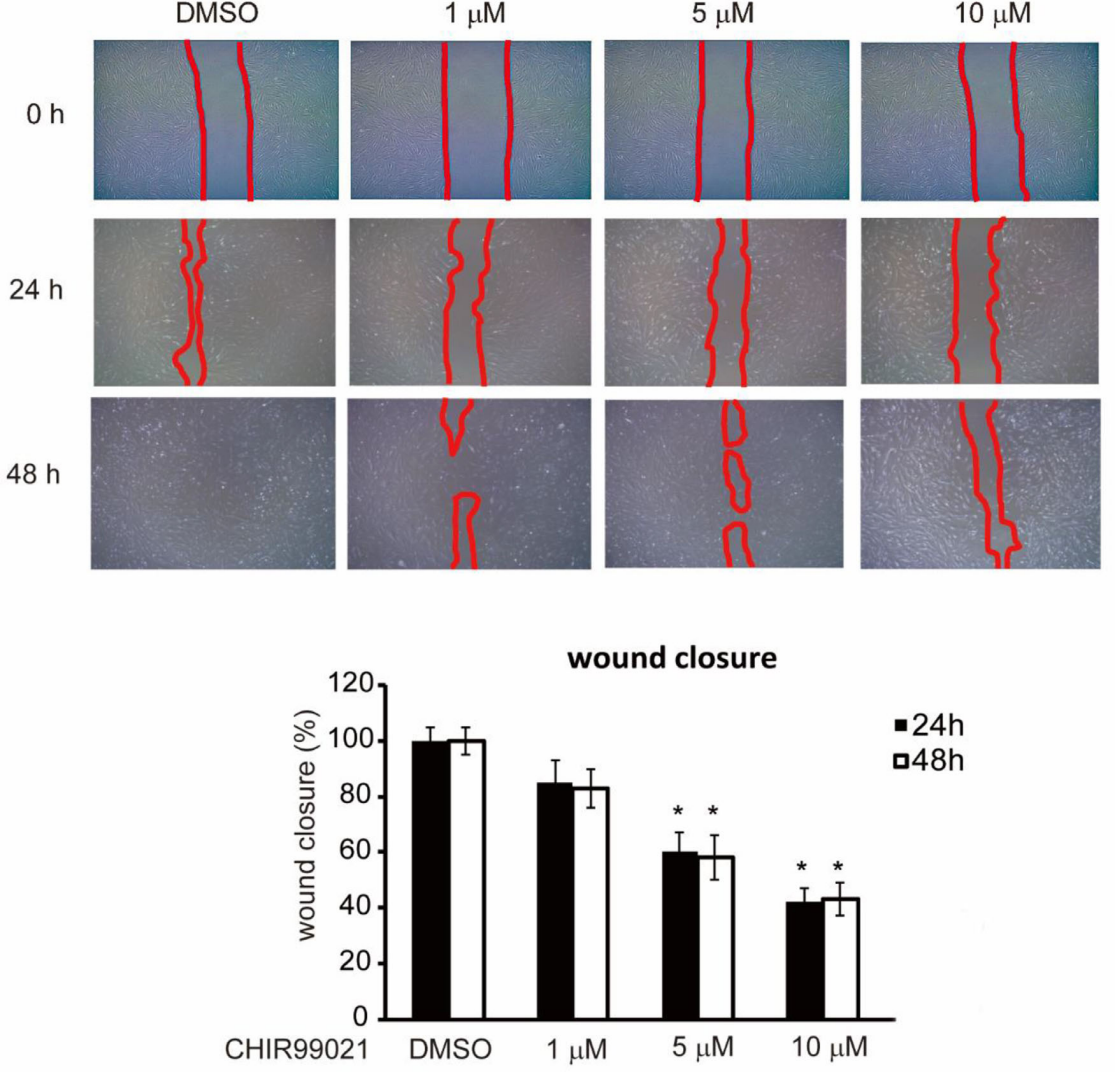
CHIR 99021 inhibits migration of the HTFs. HTFs were treated with different concentrations of CHIR 99021 (1, 5, and 10 µM) for 24 hours and 48 hours. A wound healing assay was used to evaluate cell migration. The mean wound closure ratio ± standard deviation of data from three independent repeats is presented. Red lines indicate the boundaries of the wound area. Differences between control and CHIR 99021-treated cells are indicated (*P < 0.05).

The effect of CHIR 99021 on the migration of HTFs was evaluated via wound healing assay (Fig. 6). The attenuation of cell migration was evaluated in proportion to the concentration of CHIR 99021. Relative wound closure ratios of 24-hour CHIR 99021 treatment were 85 ± 8.1%, 60 ± 7.3%, and 42 ± 5.2% for CHIR 99021 concentrations of 1 µM, 5 µM, and 10 µM, respectively. After 48 hours of treatment with CHIR 99021, the relative wound closure ratios with 1 µM, 5 µM, and 10 µM of CHIR 99021 were 83 ± 7.2%, 58 ± 8.5%, and 43 ± 6.4%, respectively.

## Discussion

In this study, we investigated the role of GSK-3β in the TGF-β–related fibrosis of HTFs using CHIR 99021, a GSK-3β inhibitor. TGF-β treatment led to a significant increase in tyrosine phosphorylation of GSK-3β (active form of GSK-3β). Treatment with CHIR 99021 significantly decreased the expression of fibronectin, collagen Iα, and α-SMA, which are characteristic molecules associated with TGF-β–related fibrosis. In addition, the results of wound healing assay showed that CHIR 99021 prevented the migration of HTFs. These results indicate that GSK-3β plays an important role in TGF-β–related fibrosis of HTFs, and inhibition of GSK-3β activation could prevent the development of excessive fibrosis in the subconjunctival space. When we compared the levels of mRNA expression of molecules associated with TGF-β–related fibrosis between normal subjects and patients with glaucoma, significantly elevated of these molecules were observed in the tenon tissue of patients with glaucoma. In addition, the GSK-3β level in the tenon tissue of patients with glaucoma also showed an increase, although it was not statistically significant. These findings demonstrate the importance of appropriately controlling bleb fibrosis after glaucoma filtration surgery. At the same time, it is postulated that GSK-3β inhibitors such as CHIR 99021 would be effective in regulating bleb fibrosis, taking into account the elevated level of GSK-3β in patients with glaucoma.

The prevention of excessive bleb fibrosis is a crucial factor for successful glaucoma filtration surgery. Steroids or nonsteroidal anti-inflammatory drugs are used for the preoperative or postoperative modulation of inflammation.^20^^,^^21^ The risk of steroid-induced increase in IOP should be always considered while using steroids. Mitomycin C and 5-fluorouracil are widely used for the postoperative maintenance of bleb function.^22^^–^^24^ As an antimetabolite, mitomycin C, which inhibits cell proliferation by intercalating DNA during the cell cycle, is often applied only once during an operation. 5-Fluorouracil is injected locally during postoperative follow-up periods, and it only affects cells in the S-phase. Regulating the proliferation of HTFs using antimetabolites is an effective method for the control of fibrosis, because it is an essential step in the wound healing process. Therefore, the use of antimetabolites during glaucoma filtration surgery has gained widespread popularity. However, there is always a risk of serious late onset postoperative complications, such as chronic hypotonia with maculopathy or an increased risk of endophthalmitis owing to the development of a thin wall of bleb. Hence, further improvements are needed for the dependable regulation of bleb fibrosis.

TGF-β is involved in many cellular processes, including growth inhibition, cell migration, invasion, epithelial–mesenchymal transition, extracellular matrix remodeling, and immune suppression.^25^ It is often chronically overexpressed in conditions of cancer, fibrosis, and inflammation, and its overexpression results in the progression of the disease via modulation of cell growth, migration, or phenotype.^26^ Therefore, the TGF-β signaling pathway has become a frequent target for the treatment of pathologic conditions. GSK-3, which consists of α and β isoforms, is activated under basal conditions and requires extracellular signaling to be inactivated, unlike most kinases.^11^^,^^27^ The activity of GSK-3 is regulated through the phosphorylation of different sites. Serine phosphorylation (S9) of GSK-3 is involved in its inactivation, whereas tyrosine phosphorylation (Y216) leads to its activation.^11^^,^^28^ A crosstalk between GSK-3 and TGF-β has been observed during the process of fibrosis in various organs. However, the function of GSK-3 in TGF-β–induced fibrosis is organ dependent. In cardiac fibroblasts, the deletion of GSK-3β led to fibrosis.^17^ In addition, excessive scarring was observed in GSK-3β knockout mouse models with an ischemic heart condition.^17^ In contrast, the pharmacological inhibition of GSK-3β attenuated the TGF-β–induced expression of fibronectin and α-SMA in pulmonary fibroblasts. In a mouse model of renal fibrosis, there was an increase in the expression and activity of GSK-3β.^12^^,^^13^ In addition, the pharmacological inhibition of GSK-3β abolished the transdifferentiation of renal fibroblasts in vivo and in vitro.^14^ Therefore, it is important to investigate whether GSK-3β is involved in the activation or inhibition of TGF-β–induced fibrosis in HTFs. As reported in the present study, the GSK-3β inhibitor CHIR 99021 effectively inhibited the expression of markers associated with TGF-β–related fibrosis of HTFs. This result suggests the possibility that GSK-3β plays a role in the process of activation of TGF-β–related fibrosis in HTFs.

It is known that Smad, in the canonical pathway of TGF-β signaling, plays an important role in fibrosis.^6^ As intermediates of TGF-β signaling, Smads are activated by serine/threonine kinases of the TGF-β receptor. Smads are classified into three types: receptor activated, mediator, and inhibitory. Of these, two receptor-activated Smads (Smad2 and Smad3) are activated in the early phase of TGF-β canonical pathway via phosphorylation of their serine residues at the C-terminus. In our study, there was a significant decrease in the phosphorylated Smad2/Smad2 and phosphorylated Smad3/Smad3 ratios when we treated CHIR 99021 with TGF-β in HTFs. Because the phosphorylated residues of Smad2 and Smad3 are Ser465/467 and Ser423/425 in the C-terminus, respectively, our result might be crucial evidence for the TGF-β pathway in HTFs being directly affected by GSK-3β inhibition. However, as suggested in a previous study on pulmonary fibrosis,^12^ there is a possibility that the TGF-β–induced cellular response in HTFs is regulated by GSK-3β–induced phosphorylation of a cAMP-responsive element-binding protein. Another possible mechanism for the crosstalk between TGF-β and GSK-3β in HTFs is through phosphorylation in the linker region. Recently, the linker region, which is located between the amino- and carboxyl-terminal Mad homology 1 (MH1) and (MH2) domains of Smad2 and Smad3, has been extensively studied because of the presence of several phosphorylation sites. These phosphorylation sites enable the crosstalk of receptor-activated Smads with other signal pathways, including GSK-3β. Several studies have reported that the phosphorylation of the linker region of Smad3 by GSK-3β is related to the inhibition of Smad3 transcription activity.^29^^,^^30^ This negative feedback can also serve as an inhibition mechanism for TGF-β–related fibrosis. Further research is needed to elucidate the exact mechanism underlying the regulation of TGF–β-related fibrosis of HTFs by GSK-3.

GSK-3 serves as a ubiquitous regulator of multiple signal pathways that are associated with the pathogenesis of various diseases, making it a promising drug target.^11^^,^^31^ The efficacy and safety of GSK-3 inhibitors have been evaluated in clinical trials for neurological diseases such as Alzheimer's disease and cognitive disorders.^32^^,^^33^ For the treatment of solid tumor or leukemia, clinical trials using GSK-3 inhibitors have been conducted or are currently in progress.^34^^,^^35^ Recently, GSK-3 inhibitor has also been used in the field of regenerative medicine for regulating the differentiation of embryonic stem cells or induced pluripotent stem cells.^36^^–^^39^ Several studies have shown that the modulation of GSK-3 induced a neuroprotective effect in retinal neurodegenerative conditions such as retinitis pigmentosa and retinal neovascularization.^40^^,^^41^ Additionally, suppression of GSK-3β using translationally relevant small interfering RNAs can enhance the survival of retinal ganglion cells.^42^ Among the various types of GSK-3 inhibitors, CHIR 99021 showed a synergistic effect with paclitaxel in the treatment of human lung cancer.^33^ In addition, a previous study has noted the possibility of using CHIR 99021 for antiadipogenic and anti-inflammatory treatment in Graves’ orbitopathy.^43^ Although there are issues regarding the selectivity for specific pathologic conditions or chronic toxicity, the scope and potential of clinical use of GSK-3 inhibitors in the medical field is increasing. However, to the best of our knowledge, no study targeting GSK-3β has been performed in the context of preventing excessive bleb fibrosis after glaucoma filtration surgery. It is clear that further studies are needed to verify our findings under different conditions, including in vivo studies, and to find the appropriate drug delivery methods necessary to sustain the effect. Even taking these limitations into account, our study may provide a foundation for further in vitro or in vivo studies to evaluate the effect of GSK-3β inhibition on prevention of excessive bleb fibrosis. In addition, considering previous studies describing the neuroprotective effect of GSK-3 modulation in retinal ganglion cells,^41^^,^^42^ the usefulness of the GSK-3 inhibitors in glaucoma treatment is expected to be very high.

In conclusion, the present study demonstrated that inhibition of GSK-3β activation by CHIR 99021 effectively downregulates the expression of proteins associated with TGF-β–induced fibrosis in primary cultured HTFs. Our study elucidates the critical role of GSK-3β in regulating TGF-β–mediated fibrosis in HTFs. The present work is a basic research showing that GSK-3β is a potential novel therapeutic target for maintaining the function of bleb after glaucoma filtration surgery. Further studies are needed to assess the potential of a GSK-3β inhibitor as a new treatment tool for increasing the success rate of glaucoma surgery.

## Supplementary Material

Supplement 1

Supplement 2

## References

[1] Weinreb RN, Aung T, Medeiros FA. The pathophysiology and treatment of glaucoma: a review. *JAMA*. 2014; 311(18): 1901–1911. 24825645 10.1001/jama.2014.3192 PMC4523637

[2] Jonas JB, Aung T, Bourne RR, Bron AM, Ritch R, Panda-Jonas S. Glaucoma. *Lancet*. 2017; 390(10108): 2183–2193. 28577860 10.1016/S0140-6736(17)31469-1

[3] Schlunck G, Meyer-ter-Vehn T, Klink T, Grehn F. Conjunctival fibrosis following filtering glaucoma surgery. *Exp Eye Res*. 2016; 142: 76–82. 26675404 10.1016/j.exer.2015.03.021

[4] Shu DY, Lovicu FJ. Myofibroblast transdifferentiation: the dark force in ocular wound healing and fibrosis. *Prog Retin Eye Res*. 2017; 60: 44–65. 28807717 10.1016/j.preteyeres.2017.08.001 PMC5600870

[5] Desmoulière A, Geinoz A, Gabbiani F, Gabbiani G. Transforming growth factor-beta 1 induces alpha-smooth muscle actin expression in granulation tissue myofibroblasts and in quiescent and growing cultured fibroblasts. *J Cell Biol*. 1993; 122(1): 103–111. 8314838 10.1083/jcb.122.1.103 PMC2119614

[6] Flanders KC. Smad3 as a mediator of the fibrotic response. *Int J Exp Pathol*. 2004; 85(2): 47–64. 15154911 10.1111/j.0959-9673.2004.00377.x PMC2517464

[7] Siriwardena D, Khaw PT, King AJ, et al. Human antitransforming growth factor beta(2) monoclonal antibody—a new modulator of wound healing in trabeculectomy: a randomized placebo controlled clinical study. *Ophthalmology*. 2002; 109(3): 427–431. 11874742 10.1016/s0161-6420(01)00997-6

[8] CAT-152 0102 Trabeculectomy Study Group, Khaw P, Grehn F, et al. A phase III study of subconjunctival human anti-transforming growth factor beta(2) monoclonal antibody (CAT-152) to prevent scarring after first-time trabeculectomy. *Ophthalmology*. 2007; 114(10): 1822–1830. 17908591 10.1016/j.ophtha.2007.03.050

[9] Pfeiffer N, Voykov B, Renieri G, et al. First-in-human phase I study of ISTH0036, an antisenseoligonucleotide selectively targeting transforming growth factor beta 2 (TGF-β2), in subjects with open-angle glaucoma undergoing glaucoma filtration surgery. *PloS One*. 2017; 12(11): e0188899. 29190672 10.1371/journal.pone.0188899 PMC5708654

[10] Yamanaka O, Liu CY, Kao WW. Fibrosis in the anterior segments of the eye. *Endocr Metab Immune Disord Drug Targets*. 2010; 10(4): 331–335. 20925651 10.2174/1871530311006040331

[11] Beurel E, Grieco SF, Jope RS. Glycogen synthase kinase-3 (GSK3): regulation, actions, and diseases. *Pharmacol Ther*. 2015; 148: 114–131. 25435019 10.1016/j.pharmthera.2014.11.016 PMC4340754

[12] Baarsma HA, Engelbertink LH, van Hees LJ, et al. Glycogen synthase kinase-3 (GSK-3) regulates TGF-β₁-induced differentiation of pulmonary fibroblasts. *Br J Pharmacol*. 2013; 169(3): 590–603. 23297769 10.1111/bph.12098 PMC3682707

[13] Jeffers A, Qin W, Owens S, et al. Glycogen synthase kinase-3β inhibition with 9-ING-41 attenuates the progression of pulmonary fibrosis. *Sci Rep*. 2019; 9(1): 18925. 31831767 10.1038/s41598-019-55176-w PMC6908609

[14] Singh SP, Tao S, Fields TA, Webb S, Harris RC, Rao R. Glycogen synthase kinase-3 inhibition attenuates fibroblast activation and development of fibrosis following renal ischemia-reperfusion in mice. *Dis Model Mech*. 2015; 8(8): 931–940. 26092126 10.1242/dmm.020511 PMC4527294

[15] Wang L, Zhu Y, Wang L, et al. Effects of chronic alcohol exposure on ischemia-reperfusion-induced acute kidney injury in mice: the role of β-arrestin 2 and glycogen synthase kinase 3. *Exp Mol Med*. 2017; 49(6): e347. 28642577 10.1038/emm.2017.76 PMC5519017

[16] Guo Y, Gupte M, Umbarkar P, et al. Entanglement of GSK-3β, β-catenin and TGF-β1 signaling network to regulate myocardial fibrosis. *J Mol Cell Cardiol*. 2017; 110: 109–120. 28756206 10.1016/j.yjmcc.2017.07.011 PMC5581678

[17] Lal H, Ahmad F, Zhou J, et al. Cardiac fibroblast glycogen synthase kinase-3β regulates ventricular remodeling and dysfunction in ischemic heart. *Circulation*. 2014; 130(5): 419–430. 24899689 10.1161/CIRCULATIONAHA.113.008364 PMC4153405

[18] Seong GJ, Hong S, Jung SA, et al. TGF-beta-induced interleukin-6 participates in transdifferentiation of human Tenon's fibroblasts to myofibroblasts. *Mol Vis*. 2009; 15: 2123–2128. 19862334 PMC2765236

[19] Livak KJ, Schmittgen TD. Analysis of relative gene expression data using real-time quantitative PCR and the 2(-Delta Delta C(T)) method. *Methods*. 2001; 25(4): 402–408. 11846609 10.1006/meth.2001.1262

[20] Breusegem C, Spielberg L, Van Ginderdeuren R, et al. Preoperative nonsteroidal anti-inflammatory drug or steroid and outcomes after trabeculectomy: a randomized controlled trial. *Ophthalmology*. 2010; 117(7): 1324–1330. 20382428 10.1016/j.ophtha.2009.11.038

[21] Liu L, Siriwardena D, Khaw PT. Australia and New Zealand survey of antimetabolite and steroid use in trabeculectomy surgery. *J Glaucoma*. 2008; 17(6): 423–430. 18794674 10.1097/IJG.0b013e31816224d8

[22] Cabourne E, Clarke JC, Schlottmann PG, Evans JR. Mitomycin C versus 5-fluorouracil for wound healing in glaucoma surgery. *Cochrane Database Syst Rev*. 2015;(11): CD00-6259, doi:10.1002/14651858.CD006259.pub2.(11):Cd006259. PMC8763343 26545176

[23] Green E, Wilkins M, Bunce C, Wormald R. 5-Fluorouracil for glaucoma surgery. *Cochrane Database Syst Rev*, 2014;(2): CD001132, doi:10.1002/14651858.CD001132.pub2.(2):Cd001132. 24554410 PMC10558100

[24] Reibaldi A, Uva MG, Longo A. Nine-year follow-up of trabeculectomy with or without low-dosage mitomycin-c in primary open-angle glaucoma. *Br J Ophthalmol*. 2008; 92(12): 1666–1670. 18782799 10.1136/bjo.2008.140939

[25] Schmierer B, Hill CS. TGFbeta-SMAD signal transduction: molecular specificity and functional flexibility. *Nat Rev Mol Cell Biol*. 2007; 8(12): 970–982. 18000526 10.1038/nrm2297

[26] Akhurst RJ, Hata A. Targeting the TGFβ signalling pathway in disease. *Nat Rev Drug Discov*. 2012; 11(10): 790–811. 23000686 10.1038/nrd3810 PMC3520610

[27] Doble BW, Woodgett JR. GSK-3: tricks of the trade for a multi-tasking kinase. *J Cell Sci*. 2003; 116(Pt 7): 1175–1186. 12615961 10.1242/jcs.00384 PMC3006448

[28] Patel P, Woodgett JR. Glycogen synthase kinase 3: a kinase for all pathways? *Curr Top Dev Biol*. 2017; 123: 277–302. 28236969 10.1016/bs.ctdb.2016.11.011

[29] Millet C, Yamashita M, Heller M, Yu LR, Veenstra TD, Zhang YE. A negative feedback control of transforming growth factor-beta signaling by glycogen synthase kinase 3-mediated Smad3 linker phosphorylation at Ser-204. *J Biol Chem*. 2009; 284(30): 19808–19816. 19458083 10.1074/jbc.M109.016667 PMC2740406

[30] Bae E, Kim SJ, Hong S, Liu F, Ooshima A. Smad3 linker phosphorylation attenuates Smad3 transcriptional activity and TGF-β1/Smad3-induced epithelial-mesenchymal transition in renal epithelial cells. *Biochem Biophys Res Commun*. 2012; 427(3): 593–599. 23022526 10.1016/j.bbrc.2012.09.103

[31] Medina M, Wandosell F. Deconstructing GSK-3: the fine regulation of its activity. *Int J Alzheimers Dis*. 2011; 2011: 479249. 21629747 10.4061/2011/479249 PMC3100567

[32] Lovestone S, Boada M, Dubois B, et al. A phase II trial of tideglusib in Alzheimer's disease. *J Alzheimers Dis*. 2015; 45(1): 75–88. 25537011 10.3233/JAD-141959

[33] Roca C, Campillo NE. Glycogen synthase kinase 3 (GSK-3) inhibitors: a patent update (2016-2019). *Expert Opin Ther Pat*. 2020; 30(11): 863–872. 32841101 10.1080/13543776.2020.1815706

[34] de Vries Schultink AH, Suleiman AA, Schellens JH, Beijnen JH, Huitema AD. Pharmacodynamic modeling of adverse effects of anti-cancer drug treatment. *Eur J Clin Pharmacol*. 2016; 72(6): 645–653. 26915815 10.1007/s00228-016-2030-4 PMC4865542

[35] Kuroki H, Anraku T, Kazama A, et al. 9-ING-41, a small molecule inhibitor of GSK-3beta, potentiates the effects of anticancer therapeutics in bladder cancer. *Sci Rep*. 2019; 9(1): 19977. 31882719 10.1038/s41598-019-56461-4 PMC6934761

[36] Sato N, Meijer L, Skaltsounis L, Greengard P, Brivanlou AH. Maintenance of pluripotency in human and mouse embryonic stem cells through activation of Wnt signaling by a pharmacological GSK-3-specific inhibitor. *Nat Med*. 2004; 10(1): 55–63. 14702635 10.1038/nm979

[37] Welham MJ, Kingham E, Sanchez-Ripoll Y, Kumpfmueller B, Storm M, Bone H. Controlling embryonic stem cell proliferation and pluripotency: the role of PI3K- and GSK-3-dependent signalling. *Biochem Soc Trans*. 2011; 39(2): 674–678. 21428960 10.1042/BST0390674

[38] Esfandiari F, Fathi A, Gourabi H, Kiani S, Nemati S, Baharvand H. Glycogen synthase kinase-3 inhibition promotes proliferation and neuronal differentiation of human-induced pluripotent stem cell-derived neural progenitors. *Stem Cells Dev*. 2012; 21(17): 3233–3243. 22642687 10.1089/scd.2011.0678

[39] Reichman S, Terray A, Slembrouck A, et al. From confluent human iPS cells to self-forming neural retina and retinal pigmented epithelium. *Proc Natl Acad Sci USA.* 2014; 111(23): 8518–8523. 24912154 10.1073/pnas.1324212111 PMC4060726

[40] Sánchez-Cruz A, Villarejo-Zori B, Marchena M, et al. Modulation of GSK-3 provides cellular and functional neuroprotection in the rd10 mouse model of retinitis pigmentosa. *Mol Neurodegener*. 2018; 13(1): 19. 29661219 10.1186/s13024-018-0251-y PMC5902946

[41] Yu L, Wu S, Che S, et al. Inhibitory role of miR-203 in the angiogenesis of mice with pathological retinal neovascularization disease through downregulation of SNAI2. *Cell Signal*. 2020; 71: 109570. 32084532 10.1016/j.cellsig.2020.109570

[42] Ahmed Z, Morgan-Warren PJ, Berry M, et al. Effects of siRNA-mediated knockdown of GSK3β on retinal ganglion cell survival and neurite/axon growth. *Cells*. 2019; 8(9): 956. 10.3390/cells8090956 PMC6769828 31443508

[43] Lee JS, Chae MK, Kikkawa DO, Lee EJ, Yoon JS. Glycogen synthase kinase-3β mediates proinflammatory cytokine secretion and adipogenesis in orbital fibroblasts from patients with Graves' orbitopathy. *Invest Ophthalmol Vis Sci*. 2020; 61(8): 51. 10.1167/iovs.61.8.51 PMC7426624 32735324

