# Supplementary material for: The Effect of CHIR 99021, a Glycogen Synthase Kinase-3β Inhibitor, on Transforming Growth Factor β-Induced Tenon Fibrosis
Source: Invest Ophthalmol Vis Sci. 2021 Dec 23;62(15):25. doi: 10.1167/iovs.62.15.25 (PMC8711002; doi:10.1167/iovs.62.15.25)

Supplementary Figure S1. Comparison of mRNA expression for the molecules related with TGF-β-induced fibrosis and GSK-3β between non glaucoma (n=5) and glaucoma (n=5) human tenon tissues using real-time PCR. Differences between non glaucoma tissues and glaucoma tissues are indicated (*$p < 0.05$).

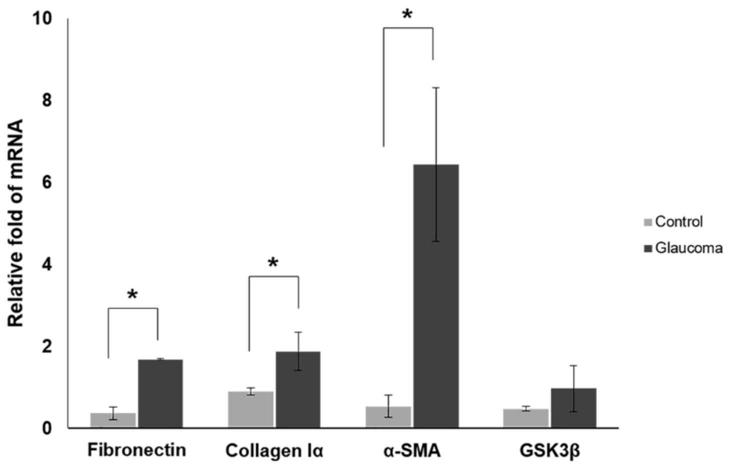

Supplement: Supplement 1 [file iovs-62-15-25_s001.pdf]
