# Supplementary material for: The Effect of CHIR 99021, a Glycogen Synthase Kinase-3β Inhibitor, on Transforming Growth Factor β-Induced Tenon Fibrosis
Source: Invest Ophthalmol Vis Sci. 2021 Dec 23;62(15):25. doi: 10.1167/iovs.62.15.25 (PMC8711002; doi:10.1167/iovs.62.15.25)
Supplement: Supplement 2 [file iovs-62-15-25_s002.pdf]

Supplementary Material

Supplementary Table S1. Characteristics of patients.

	Age	Sex	Surgery	Systemic disease	Pre-operative ophthalmic medication	Ophthalmic medication duration (months)
**Non-glaucoma**						
	62	M	Cataract	None	None	N/A
	57	F	Cataract	HTN	None	N/A
	59	F	Cataract	None	None	N/A
	70	F	Cataract	None	None	N/A
	64	F	Cataract	None	None	N/A
**Glaucoma (type)**						
OAG	60	M	Cataract	HTN	Latanoprost	31
ACG	72	F	Cataract	HTN	Betaxolol<br>Latanoprost	15
OAG	86	F	Cataract	None	Tafluprost	42
OAG	68	F	Cataract	HTN	Latanoprost	75
OAG	67	F	Cataract + Ahmed	None	Tafluprost<br>Timolol<br>Dorzolamide<br>Brimonidine	132

OAG, open-angle glaucoma; ACG, angle-closure glaucoma; HTN, hypertension

Supplementary Table S2. Primer sequences for RT-PCR.

**Primer**	**Sequence (5'-3')**	**Direction**
GAPDH	ATG GGG AAG GTG AAG GTC G	Forward
	GGG GTC ATT GAT GGC AAC AAT A	Reverse
GSK-3β	TCA AGG CAC ATC CTT GGA CAA	Forward
	TGC ACA AGC TTC CAG TGG TGT	Reverse
Fibronectin	TCG AGG AGG AAA TTC CAA TG	Forward
	ACA CAC GTG CAC CTC ATC AT	Reverse
Collagen Iα	CGC ATG AGC GGA CGC TAA CC	Forward
	TTC CTC TTG GCC GTG CGT CA	Reverse
α-SMA	GAC CCT GAA GTA CCC GAT AGA AC	Forward
	GGG CAA CAC GAA GCT CAT TG	Reverse
